# Qualitative study on the use and maintenance of long-lasting insecticidal nets (LLINs) in Bouaké (Côte d’Ivoire), 17 months after the last mass distribution campaign

**DOI:** 10.1186/s12936-022-04243-1

**Published:** 2022-07-29

**Authors:** Gnagoran Kouakou Daniel N’Guessan, Fangala Hamidou Coulibaly, Antoine Marc Gaby Barreaux, Roseline Josée Yapo, Kouassi Arsène Adou, Emmanuel Tia, Florence Fournet

**Affiliations:** 1 Centre d’Entomologie Médicale Et Vétérinaire—Université Alassane Ouattara, Bouaké, Côte d’Ivoire; 2 IRD, CNRS), Institut de Recherche Pour Le Développement (IRD), MIVEGEC (Univ. Montpellier, Montpellier, France; 3 Centre Suisse de Recherches Scientifiques en Côte d’Ivoire, Abidjan, Côte d’Ivoire; 4 Bristol Veterinary School, University of Bristol, Bristol, UK; 5 INTERTRYP (Univ. Montpellier, CIRAD, IRD), Montpellier, France; 6 Institut de Géographie Tropicale—Université Félix Houphouët-Boigny, Abidjan, Côte d’Ivoire; 7 Institut Pierre Richet—Institut National de Santé Publique (INSP), Bouaké, Côte d’Ivoire

**Keywords:** Malaria, LLINs, Usage, Maintenance, Washing, Côte d’Ivoire

## Abstract

**Background:**

The use of long-lasting insecticide-treated nets (LLINs) is one of the main malaria prevention method promoted by the World Health Organization (WHO) in Côte d'Ivoire. LLIN-coverage has reached 95% since 2015 and nearly 16 million LLINs were distributed in 2017. Despite these efforts, malaria incidence at the national level remains high (120‰ in 2012 to 164‰ in 2017) although this could be partly explained by increased screening efforts. This study aimed at determining what preventative measures were used against mosquito bites, as well as LLIN maintenance practices used by the inhabitants of the city of Bouaké, capital city of the Gbêkê region with a malaria incidence of 257‰ in 2017.

**Methods:**

A descriptive qualitative investigation took place in Bouaké, in four neighbourhoods that were selected through purposive sampling based on their social composition. Data were collected using an interview guide based on convenience sampling.

**Results:**

The results of the study reveal that LLINs are the most reported used malaria prevention measure (66.4%). Environmental health (28.8%) came second in their declarations, smoke coils (23.5%) third and aerosol cans (18.8%) last. The percentage of respondents who answered that they had slept under an LLIN the previous night was 53%. 57.7% reported that they wash their LLINs, 12.1% that they do not wash them, and 4% that they replace dirty LLINs with new ones. The LLINs washing methods described by the respondents did not comply with the WHO recommendations and there was no mention of LLINs repairs.

**Conclusion:**

Despite mass distributions of LLINs in Côte d'Ivoire, this key malaria control tool remains under-used by the population. Regarding LLIN maintenance, more than half of the population reports that they wash their nets while not complying with recommended practices or repairing them.

## Background

Malaria is a disease caused by *Plasmodium* parasites, transmitted to humans by the bite of a female *Anopheles* mosquito. Malaria is a leading cause of morbidity and mortality in the 91 countries where it is endemic [1]. In Côte d'Ivoire, malaria remains the primary reason for consultation in health facilities despite a national coverage of long-lasting insecticidal nets (LLINs) estimated at 95% since 2015 [2]. The number of malaria related cases and deaths recorded in 2017, are 4,032,381 and 3,886, respectively [3]. In 2018, the Regional Health Directorate of Gbêkê, a region located in the centre of the country, recorded 206,378 cases and 106 deaths related to malaria for the capital city of Bouaké. Children under 5 years of age in this city paid the highest price with 58,906 cases and 84 deaths.

Malaria control combines the control of the parasite and of the vector. The former is based on preventive or curative drug treatments and the latter aims at protecting populations from mosquito bites and reducing the intensity of local transmission [4–7]. Vector control reduces human-vector contact, vector longevity and mosquito densities. It is mainly based on the distribution of LLINs and indoor residual spraying (IRS) [5, 8–10].

At present, LLINs play a very important role in the fight against malaria worldwide by providing a physical and chemical barrier to mosquitoes and they are one of the most effective tools to prevent malaria transmission [9, 11, 12]. In malaria-prone areas, many countries have adopted a LLIN universal coverage policy, as a LLIN coverage of at least 80% should indeed reduce the malaria burden [1, 13–16]. According to the 2016 Multiple Indicator Cluster Survey (MICS), 75.1% of households in Côte d'Ivoire have at least one LLIN and 50.1% of people slept under LLINs the night before the survey.

Well-maintained LLINs can retain their physical integrity and effectiveness for at least three years [6, 17–19]. To do so, they should be washed with cold water and mild soap, using gentle strokes. They should not be washed more than once every three months and should be dried in the shade. They should also be repaired immediately when they are punctured. It is advised to tie them up when they are not in use [18, 20, 21].

Factors such as tears, dirt, or improper washing practices may reduce their effectiveness and increase users' risk of contracting malaria [15, 20, 22, 23]. LLINs with holes in them greatly reduce personal protection [24]. Another study in Kenya showed that repeated washing of LLINs over short intervals leads to their biological ineffectiveness [23] demonstrating the importance of following the recommendations. It was confirmed by a study in Benin for Olyset^®^ and PermaNet^®^. However, some nets may be more resistant to improper washing as in the same study the LifeNet^®^ net remained highly effective against *Anopheles gambiae *sensu stricto (*s.s.*) after repeated washing [25].

Research on community-based LLIN washing practices in Benin has shown that people use traditional soap or local soap to wash their LLINs [26]. LLINs washed with traditional soap and let to dry in the sun lose their effectiveness quickly compared to those washed with local soap and dried in the shade. A previous study on user perceptions and effectiveness of LLINs in Côte d'Ivoire [27], showed that the use of industrial soap powder and a moderate frequency of washing with tap water maintained the effectiveness of LLINs. An evaluation of the use and maintenance of LLINs conducted as part of an integrated control strategy in a malaria endemic area in the Brazilian Amazon, showed that LLINs distributions were not combined with educational strategies conclusive to long-term use [28].

Under-utilization of LLINs, as well as poor maintenance practices could, therefore, reduce LLINs effectiveness at the community level. These factors may explain why, despite the large-scale distribution of LLINs, the number of cases and deaths related to malaria remains high, especially in Côte d'Ivoire and particularly in the Gbêkê region. The objective of this qualitative study was to determine the preventative measures used by the population against mosquitoes as well as the maintenance and use of LLINs in Bouaké.

## Methods

The aim was to understand the knowledge, attitudes, and practices of LLINs use and maintenance regarding the information received. A descriptive qualitative investigation was performed, and social representations were collected using the discursive production method [29–31]. Interviews were conducted on February 26th to 28th, 2019.

### Study sites

**Fig. 1:**
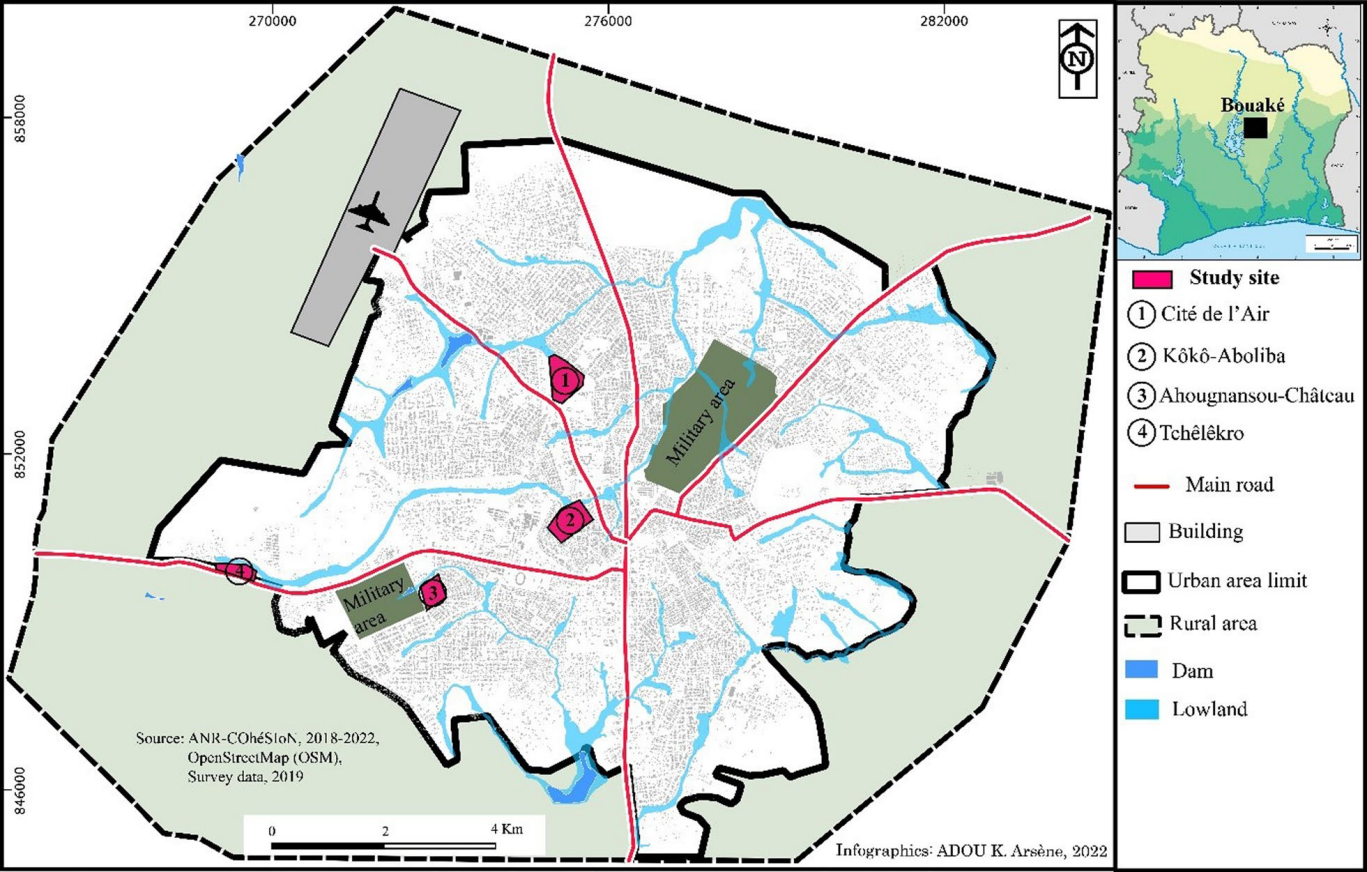
The city of Bouaké and the location of the four study districts

The study took place in the city of Bouaké (7°4’00’ north, 5°01’59’ west), capital of the Gbêkê region in central Côte d'Ivoire. Four neighbourhoods were selected by reasoned choice based on their contrasting social composition and housing (Fig. 1):

- Cité de l'Air is a recent residential neighbourhood, sparsely populated with modern houses (some still in construction) and bare land covered with scrub.
- Kôkô Aboliba is an old and working-class neighbourhood located downtown and marked by the presence of lowlands. It is characterized by a high population density. It is made up of a population of varied socio-linguistic origin.
- Ahougnansou Château is a more recent working-class district, located in the third urban extension ring, which began between 1970 and 1980. The built environment is relatively dense with a mix of modern and older housing. There are numerous lowlands.
- Tchèlèkro is a peripheral neighbourhood with a rural characteristic. It is surrounded by a lowland with rice cultures and market gardening. The neighbourhood is growing in density by expanding into cultivated land. The population of this neighbourhood is less cosmopolitan than that of Kôkô Aboliba.

### Data collection

Data was collected using an interview guide and the personal interview method (interviewer facing the respondent) [32]. The interview guide consists of 12 questions: three closed questions on the socio-demographic characteristics of the respondents, two open questions on their knowledge of malaria vector control tools, three closed questions on the use of LLINs, and four questions, two of which are closed, on LLINs maintenance. The non-probability sampling technique was used to construct the sample. In each neighbourhood, the sample was defined on the basis of the saturation of the collected information. Individuals were randomly selected from households and workplaces based on their availability.

### Data analysis

The Sphinx V5 software was used to calculate the relative frequencies of the LLINs usage and maintenance indicators. It was also used to extract the content of text variables from the answers to the open-ended questions (verbatim). The excerpts were then presented in lists organized by response category. Content analysis was then used to synthesize the information provided by the open-ended questions and to make sense of it in relation to the context of the study.

## Results

### Characteristics of the study population

A total of 149 individuals, 83 men and 66 women, who received LLINs at health centres and during free distribution campaigns were surveyed. The age of the respondents ranged from 15 to 73 years old. Two-thirds were 15 to 39 years old, and the remaining third were 40 to 73 years old. More than a quarter of the respondents have never attended school and almost 21% went to university. There was an important heterogeneity among the neighbourhoods (Table 1).

**Table 1:** Educational level of the respondents

Educational level	Kôkô Aboliba n (%)	Cité de l’Air n (%)	Tchèlèkro n (%)	Ahougnansou Château n (%)
None	10 (38.5)	15 (30)	9 (41)	5 (9.8)
Primary	4 (15.4)	6 (12)	3 (13.6)	10 (19.6)
Middle school	7 (26.9)	2 (4)	6 (27.3)	9 (17.6)
Secondary	4 (15.4)	14 (28)	1 (4.5)	13 (25.5)
University	1 (3.8)	13 (26)	3 (13.6)	14 (27.5)

Source: Field survey data, 2019

### Means of protection against malaria cited by respondents

**Fig. 2:**
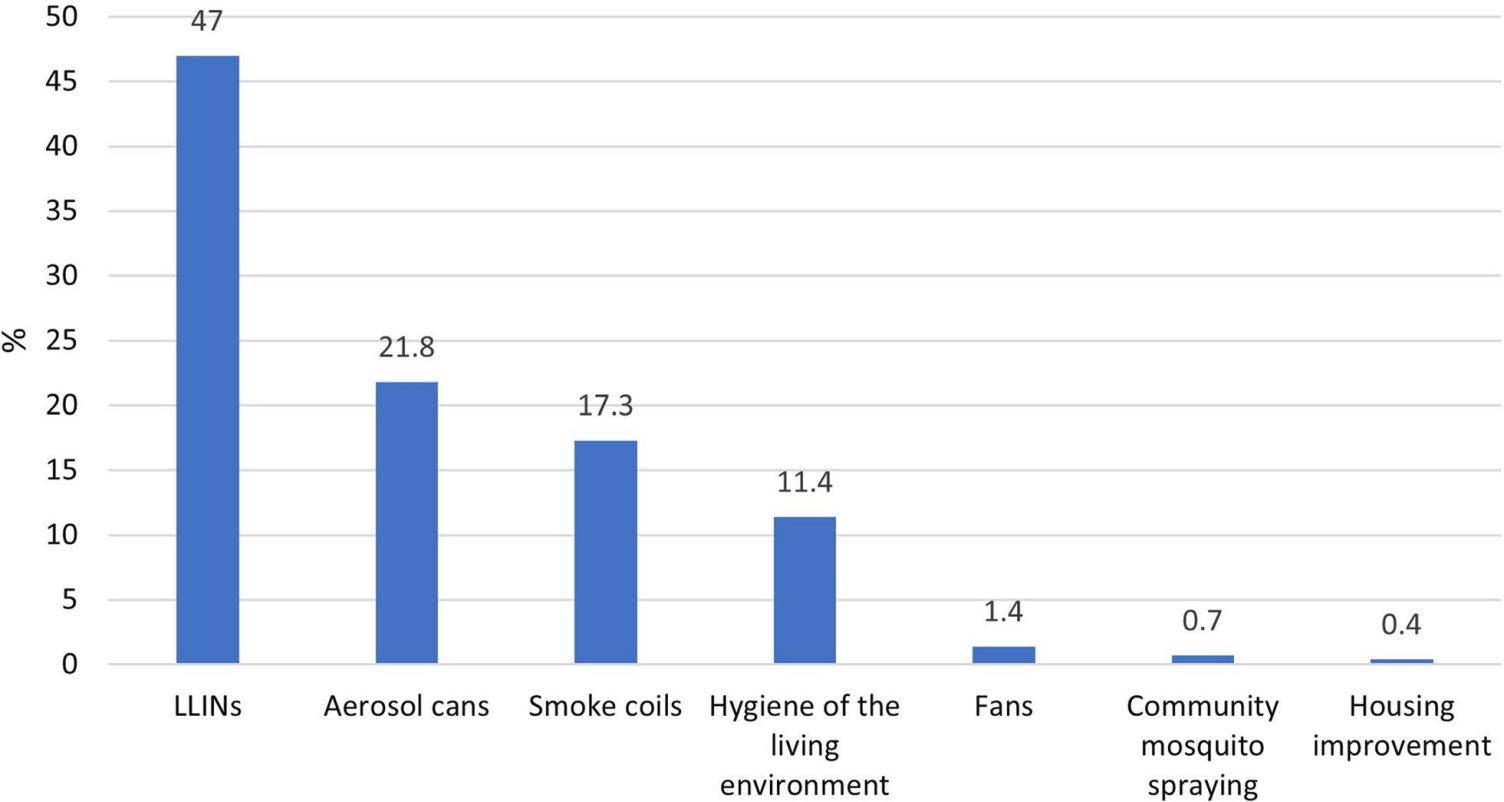
Protective methods mentioned by respondents to prevent malaria. Source: Field survey data, 2019. *A respondent could cite several protective methods

Respondents mentioned a range of malaria prevention methods, among which LLINs were the most frequently mentioned (47%) (Fig. 2).

### Reported malaria vector control tools and methods used by the respondents

**Fig. 3:**
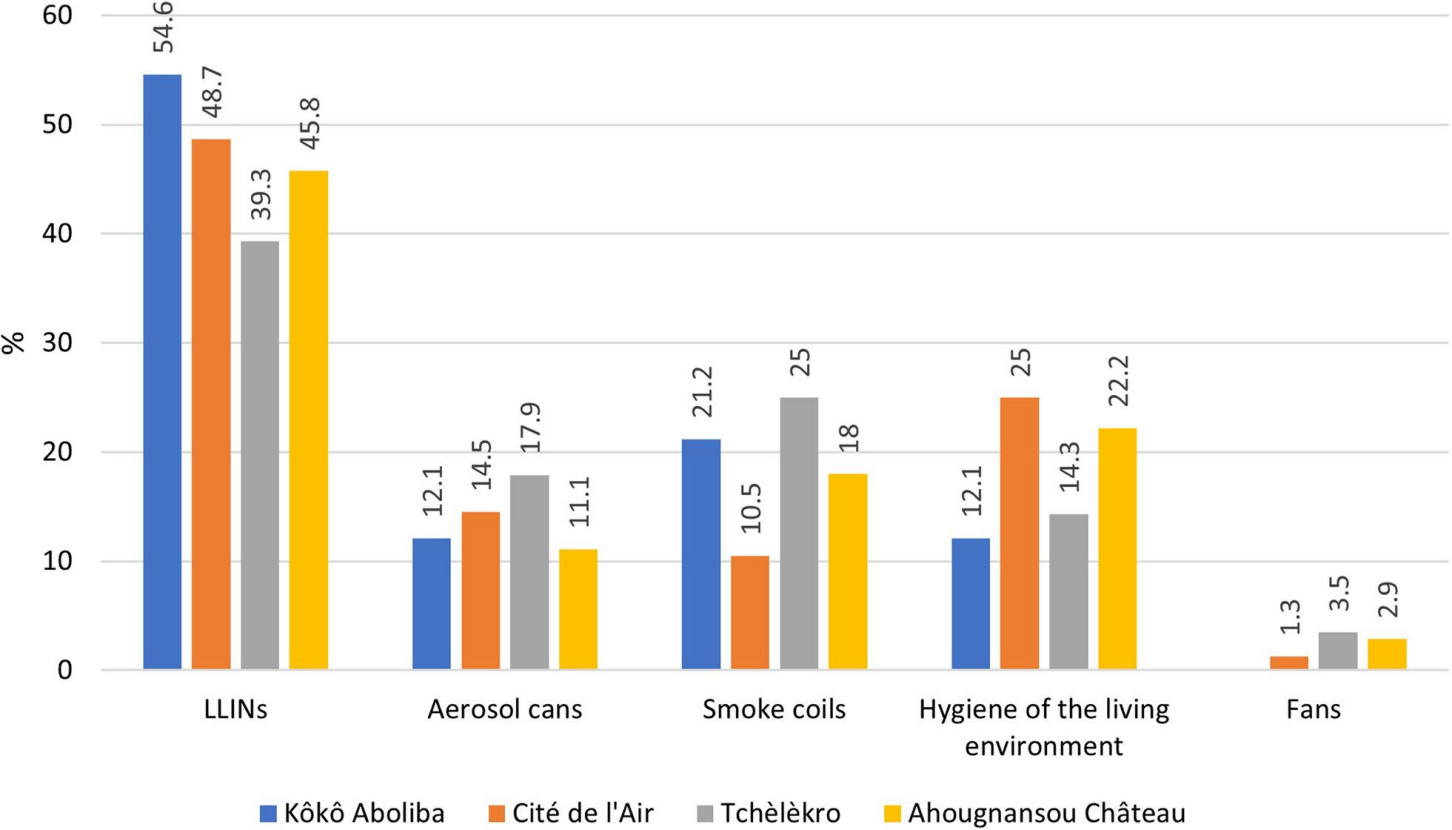
Reported malaria vector control tools and methods use by neighbourhoods. Source: Field survey data, 2019. *A respondent could cite several vector control tools and methods

LLINs were the more commonly reported vector control tools used against malaria in each neighbourhood. The hygiene of the living environment was less used in Tchèlèkro and Kôkô Aboliba compared to Cité de l’Air and Ahougnansou. A low use of smoke coils was observed at Cité de l’Air. Aerosol cans were used to a greater extent in Cité de l’Air and Tchèlèkro (Fig. 3).

### Willingness to sleep under an LLIN

**Fig. 4:**
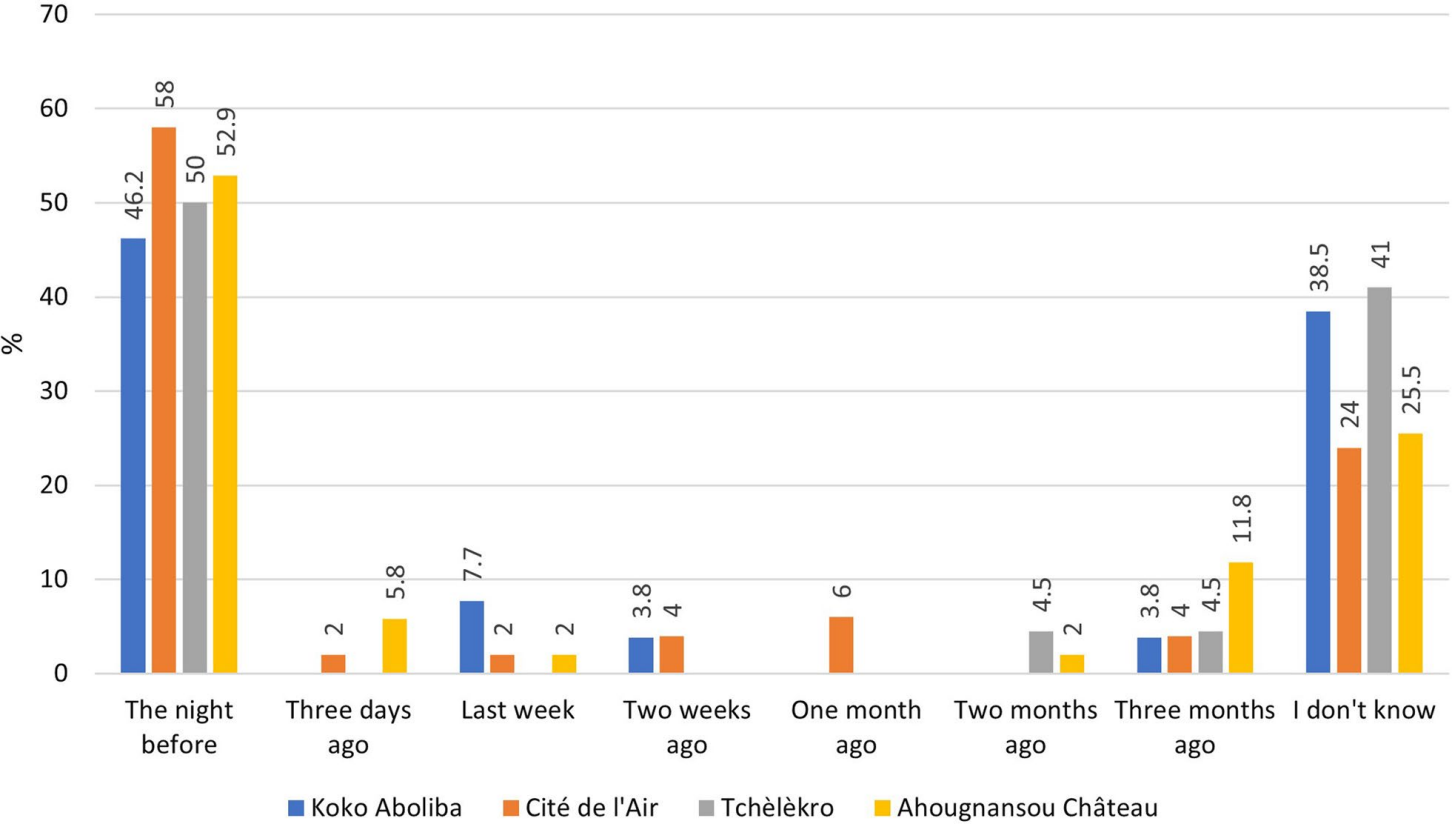
Reported LLIN use by neighbourhood. Source: Field survey data, 2019

Overall, 87.9% (131/149) of respondents said they were in favour of using an LLIN but only 53% had used it the night before the survey (Fig. 4). Koko Aboliba had the lowest use of LLINs the previous night (46.2%) and Cité de l’Air the highest (58%). Whatever the neighbourhood, 29.5% of respondents said they could not remember the last time they had slept under an LLIN, with the highest proportion in Kôkô Aboliba and Tchèlèkro (38.5% and 41%, respectively). 47% of people having received nets reported not using them regularly.

### Attitude of the respondents towards washing their LLINs

Of the populations surveyed, 87.2% (130/149) knew that LLINs must be maintained to keep them effective. Only 14.8% (22/149) of respondents mentioned having received training on how to maintain LLINs. Respondents' knowledge about LLINs maintenance was dominated by their knowledge and representation of household laundry care.

**Fig. 5:**
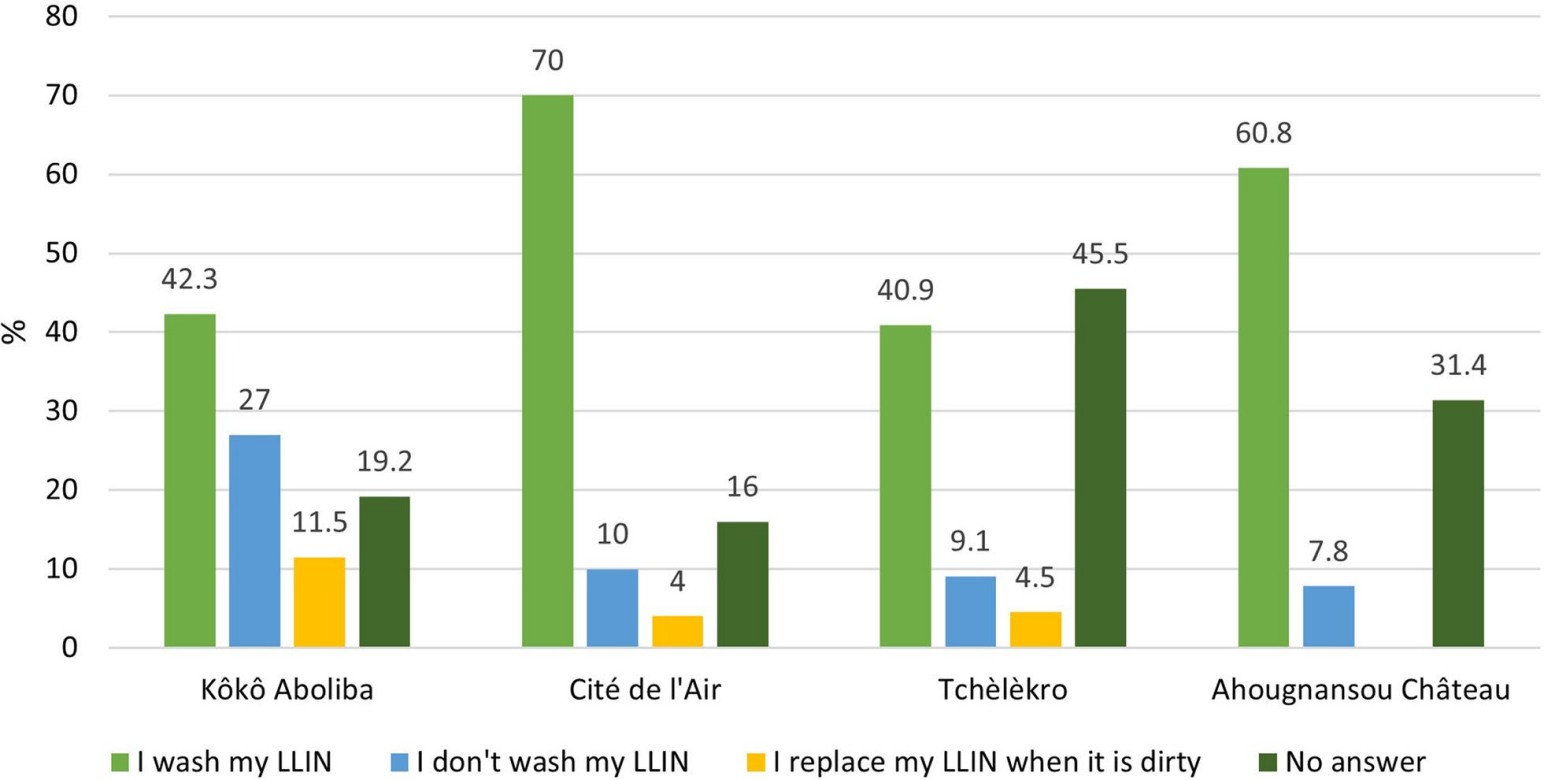
Reported LLIN washing behaviour by neighbourhood. Source: Field survey data, 2019

Overall, 57.7% (86/149) of respondents reported washing their LLINs, 12.1% (18/149) did not wash them, and 4% (6/149) replaced their dirty LLINs with new ones (Fig. 5). 26.2% of the individuals surveyed did not answer questions about the maintenance of their LLINs with the highest proportion in Tchèlèkro. The proportions of people who washed their LLINs were higher in Ahougnansou Château (60.8%) and Cité de l'Air (70%).

### Detergents used by the respondents to wash the LLINs

Four types of detergents were used by the respondents who washed their LLINs. These were OMO (industrial powdered detergent), Savon de Marseille (traditional soap), bleach, and Kabakourou (local soap). The study revealed that the detergents were either used individually or in combination (OMO + Savon de Marseille, OMO + bleach).

**Fig. 6:**
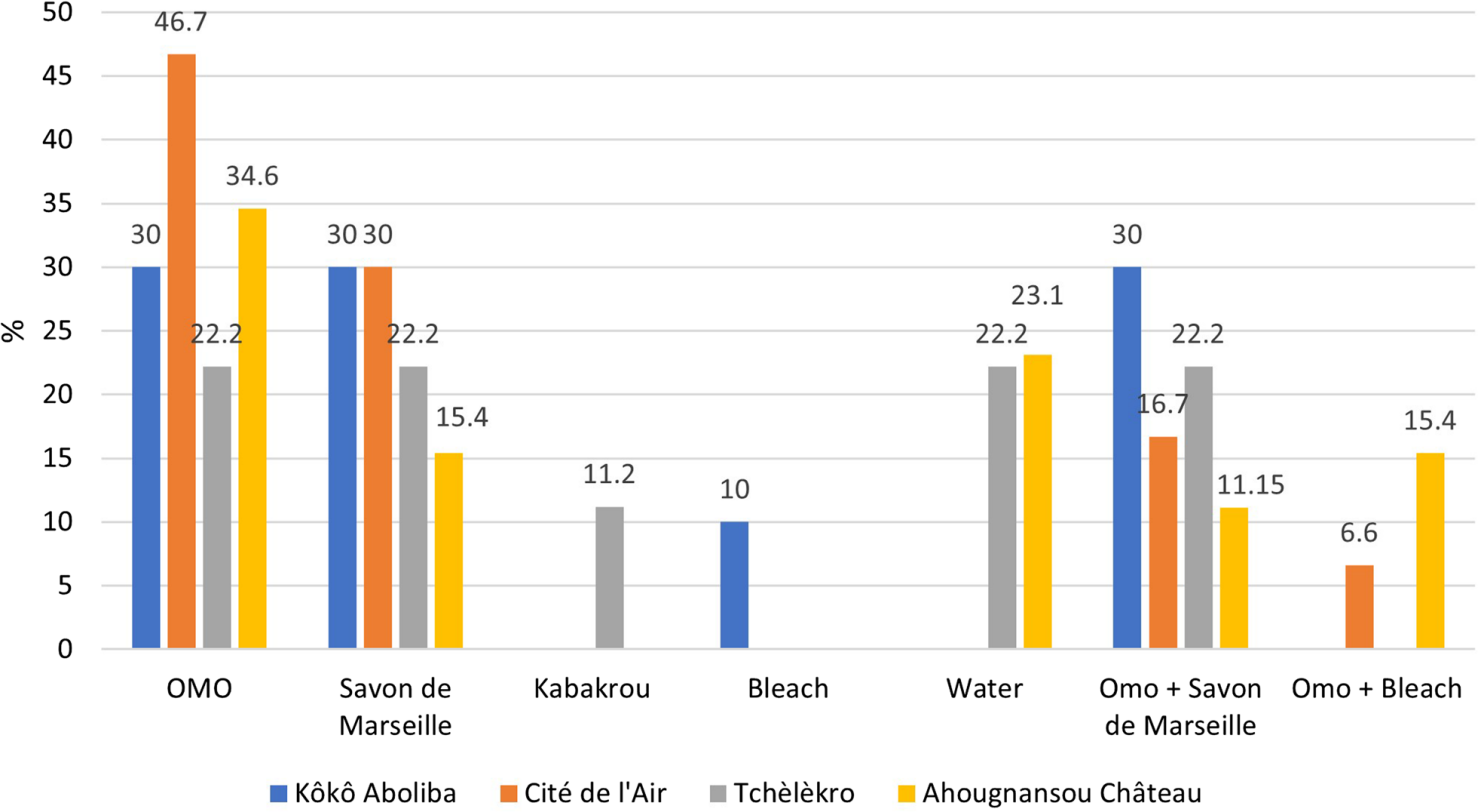
Detergents reported for washing LLINs by neighbourhood. Four types of detergents were used by the respondents who washed their LLINs. These were OMO (industrial powdered detergent), Savon de Marseille (traditional soap), bleach, and Kabakourou (local soap). The study revealed that the detergents were either used individually or in combination (OMO + Savon de Marseille, OMO + bleach). Source: Field survey data, 2019

Overall, the most used detergent was OMO 37.3% (28/75), followed by Savon de Marseille 24% (18/75) and the combination of OMO + Savon de Marseille 7.3% (13/75) (Fig. 6). 10.7% (8/75) of respondents washed their LLINs without using any detergent. The use of bleach alone is only observed in Kôkô Aboliba (10%) and the use of Kabakourou alone only in Tchèlèkro (1.2%). The use of detergent combinations represents 25.3% of the declarations.

### LLINs washing frequency reported by the respondents

**Fig. 7:**
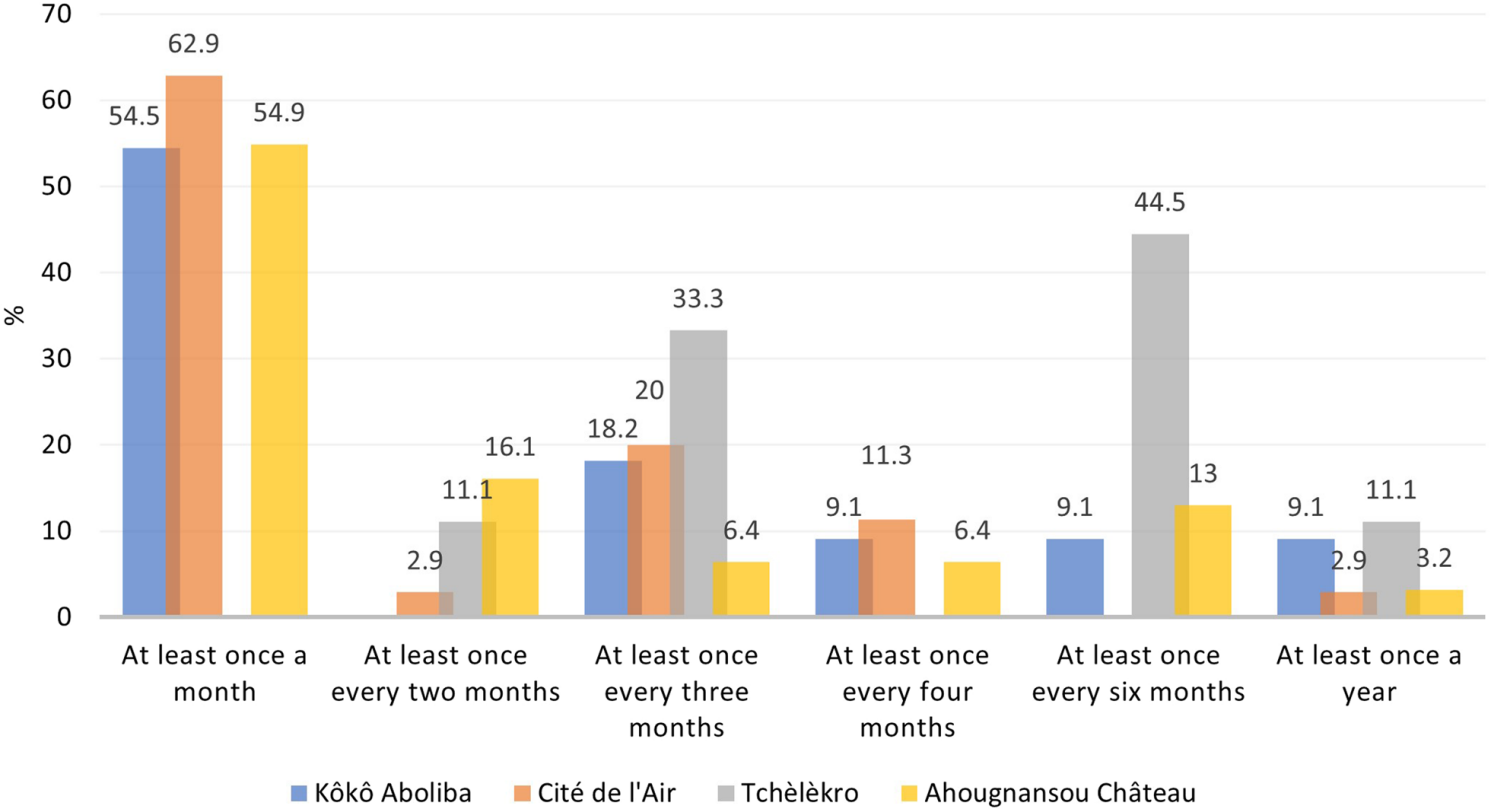
LLIN washing frequency reported by neighbourhood. Source: field survey data, 2019

Among the 57.7% of respondents who wash their LLINs, the frequency of washing varied by neighbourhood (Fig. 7). The majority of respondents 52.3% (45/86) reported washing their LLIN at least once a month. However, the respondents from Tchèlèkro reported washing their LLIN only every 6 months.

Some excerpts from the respondents’ open discourse on the maintenance of the LLIN.

*"I wash my net when it is dirty. I wash it like I wash my clothes. I wash it once a month with OMO". (Woman, 28-year-old, Koko Aboliba)*

*"I wash with OMO, rinse and dry in shade three time a year". (Man, 20-year-old, Cité de l’Air)*

*"I wash with OMO, and by hand every six months". (Woman, 24-year-old, Cité de l’Air)*

Source: field survey data, 2019.

## Discussion

Respondents in the different neighbourhoods reported using several vector control methods to protect themselves from mosquito bites. The LLIN was the most used method (47.3%). This low percentage clearly shows that people in Bouaké underuse LLINs. Finally, two trends could be found: a "traditional" trend in Tchèlèkro and Kôkô Aboliba, and a "modern" trend in Ahougnansou Chateau and Cité de l'Air. In Cité de l'Air and Ahougnansou Chateau, the behaviors seemed to be rather in line with the World Health Organization (WHO) recommendations concerning the recognition of LLINs as an effective preventative method against mosquito bites, and the need to sleep under LLINs and wash them. These two neighbourhoods, reported a higher educational level and have a higher urbanization degree compared to Tchèlèkro and Kôkô Aboliba.

Respondents also consider the hygiene of their living environment as a method of protection against mosquito bites. It is less used in traditional neighbourhoods (Kôkô Aboliba and Tchèlèkro) compared to more modern ones (Cité de l'Air and Ahougnansou Château). Since in modern neighbourhoods, most of the respondents have at least a high school education, it can be suggested that socioeconomic level and/or education play a role in awareness of hygiene as an effective method to prevent malaria infections. Indeed, unhealthy environments appear to favour the exposure to mosquito bites. This component must, therefore, be taken into account in malaria vector control, as it is already the case for *Aedes* control [9].

There was a moderate reported use of LLINs the night before the survey in each neighbourhood. This may be due to the lack of community-based health education systems that would encourage people to sleep under LLINs. Only 53% of all respondents slept under an LLIN the night before the survey even though 87% were in favour of using them. This rate is well below the 80% threshold recommended by the WHO, and is roughly equal to the 50.1% reported by the MICS 2016 data [33] or the 51% reported in 2020 during the evaluation of an LLIN distribution program in western India [19]. In Nigeria, however, 92% of respondents reported having slept under an LLIN the night before the survey [34]. The results obtained in the present study indicate that 30.2% of respondents could not remember the last time they had slept under an LLIN, which makes it clear that LLINs are not frequently used by these persons. As previously hypothesized in another study [28], LLINs were distributed without an educational strategy allowing for their long-term use. The lack of LLINs use can often be explained by several hindering factors such as the feeling of suffocation, the feeling of heat and low densities of nuisance mosquitoes [35]. In the case of the four districts studied, the lack of LLIN use may firstly be explained by the use of alternative malaria vector control tools within the populations.

Regarding LLIN maintenance, 87.2% of the respondents know that LLINs must be maintained to assure their long-term effectiveness but only 14.8% of respondents declared having received training on their maintenance. 26.2% of respondents did not answer the question about their attitudes towards washing LLINs. This can be understood either as embarrassment from not washing their LLINs, not being able to afford washing them, or as a lack of awareness about washing methods. To wash their LLINs, respondents use different types of detergents: traditional soap (kabakrou), Savon de Marseille, industrial powder detergent (OMO) and some use bleach. Most respondents wash their LLINs at least once a month. The heterogeneity in the discourses collected on LLINs washing in the different neighbourhoods of our study indicates that the correct washing practices are unknown to the population. This was observed in the four neighbourhoods, which enables us to affirm that awareness of LLINs washing is not associated with the population's living standard and/or education. The practices of the respondents in regard to LLINs maintenance show that their knowledge is dominated by their representation of the maintenance of household linen. This is not suitable for LLINs that should be washed with cold water and mild soap in gentle strokes to maintain their physical integrity and effectiveness for at least three years [6, 17–19].

Similar results were obtained in Ilorin Kwara State, Nigeria [34] showing that 88% of the washing were inappropriate. The present study reveals poor maintenance practices for LLINs, in contrast to a previous study in western Kenya [36]. Six respondents said that they do not wash dirty LLINs but replace them with new ones. Replacing LLINs once they are dirty may be due to the fact people are not informed about maintenance, do not say what they actually do, or have enough LLINs to replace the dirty ones. These results raise questions about the effectiveness of the mass LLINs distribution strategy [28].

## Conclusion

Despite the favourable attitude of the population to sleeping under an LLIN, this effective tool against malaria remain underused. The different washing methods described show that people are unaware of the WHO recommendations for proper washing of LLINs. It also appears that people's knowledge of how to maintain their LLINs is incomplete, as repairing and knotting were not mentioned in the responses to the open-ended question on LLIN maintenance. Under-use and lack of knowledge of good maintenance practices for this key malaria control tool can be interpreted as one of the main factors that explains why, despite high coverage of LLINs, the number of malaria cases and deaths remains high in Bouake. Identifying the factors limiting the proper use, washing and maintenance of LLINs, and consequently the parameters that could enhance LLIN use, will allow the establishment of community-based health education system to sensitize and train people in the use and maintenance of their LLINs. This could largely contribute to the reduction of malaria transmission in Côte d’Ivoire and more widely.

## Data Availability

All data generated or analysed during this study are included in this published article.

## Abbreviations

IRS: Indoor residual spraying
LLIN: Long-lasting insecticide-treated net
MICS: Multiple indicator cluster survey
WHO: World Health Organization
